# Supplementary material for: Simulation-based training using a vessel phantom effectively improved first attempt success and dynamic needle-tip positioning ability for ultrasound-guided radial artery cannulation in real patients: An assessor-blinded randomized controlled study
Source: PLoS One. 2020 Jun 11;15(6):e0234567. doi: 10.1371/journal.pone.0234567 (PMC7289374; doi:10.1371/journal.pone.0234567)
Supplement: S1 Table — (DOCX) [file pone.0234567.s001.docx]

**Supplement table 1.** Quiz for background knowledge on ultrasound-guided radial artery cannulation.

| **Answer the question Yes or No** |  |
| --- | --- |
| 1. **The clinician should cover the ultrasound transducer with a sterile sheath before wearing the sterile gloves.** | 0 = No 1 = YES |
| 1. **The clinician should place the ultrasound machine directly across from the operator to establish the best ergonomic position for the procedure.** | 0 = No 1 = YES |
| 1. **Once the target artery is identified, depth and brightness of the ultrasound image should be optimized by adjusting the ultrasound machine. This makes the procedure easy.** | 0 = No 1 = YES |
| 1. **If the target artery is in the middle of the ultrasound image, you do not need to align the ultrasound screen image direction with the ultrasound transducer direction.** | 0 = No 1 = YES |
| 1. **Repeated failure of ultrasound-guided radial artery cannulation do not increase complication rate.** | 0 = No 1 = YES |
| 1. **It is necessary to scan the artery’s size, whether it is branched before the puncture to find a suitable location for the procedure.** | 0 = No 1 = YES |
| **7) In order to get a better image, press the skin with the ultrasound transducer as possible.** | 0 = No 1 = YES |
| **8) Before vascular cannulation, it is essential to verify that the catheter tip (“bright white dot”) is in the midline of the anterior wall of the target artery.** | 0 = No 1 = YES |
| **9) If the catheter tip is not identifiable in the ultrasound image, it is necessary to slide the ultrasound transducer back and forth to find it before advancing the catheter.** | 0 = No 1 = YES |
| **10) If you think the catheter was successfully inserted into the radial artery, connect it to the pressure monitor line and confirm it by the arterial wave form.** | 0 = No 1 = YES |
| **Total Sum** | **/ 10** |
