# Supplementary material for: Simulation-based training using a vessel phantom effectively improved first attempt success and dynamic needle-tip positioning ability for ultrasound-guided radial artery cannulation in real patients: An assessor-blinded randomized controlled study
Source: PLoS One. 2020 Jun 11;15(6):e0234567. doi: 10.1371/journal.pone.0234567 (PMC7289374; doi:10.1371/journal.pone.0234567)
Supplement: S2 Table — (DOCX) [file pone.0234567.s002.docx]

**Supplement Table 2.** 5-point scale for assessing the change in self confidence after the simulation training set.

| **Please score your change of self-confidence in ultrasound-guided radial artery cannulation after the simulation training set.** | |
| --- | --- |
| 1. The self-confidence decreased after the simulation training set. 2. The self-confidence did not change after the simulation training set. 3. The improvement in self-confidence is unclear after the simulation training set. 4. The self-confidence improved insufficiently after the simulation training set. 5. The self-confidence improved sufficiently after the simulation training set. | |
| **Total Sum** | **/ 5** |
