# Supplementary material for: Simulation-based training using a vessel phantom effectively improved first attempt success and dynamic needle-tip positioning ability for ultrasound-guided radial artery cannulation in real patients: An assessor-blinded randomized controlled study
Source: PLoS One. 2020 Jun 11;15(6):e0234567. doi: 10.1371/journal.pone.0234567 (PMC7289374; doi:10.1371/journal.pone.0234567)
Supplement: S3 Table — (DOCX) [file pone.0234567.s003.docx]

**Supplement table 3.** Checklist for procedures of ultrasound-guided radial artery cannulation

| **1. Skilled in working with an ultrasound machine** | |
| --- | --- |
| 1. **Did the participant place the ultrasound in front of them?** 2. **Did the participant hold the ultrasound in one’s non-dominant hand?** 3. **Did the participant align the ultrasound transducer with the ultrasound screen direction?** 4. **Is the hand firmly rested on the patient’s wrist to keep the ultrasound transducer stable?** 5. **Can the participant modify depth and brightness of the ultrasound screen?** 6. **Can differentiate artery and vein on the ultrasound screen (By color doppler image or by compressing the structure)?** 7. **Is the ultrasound transducer perpendicular to the radial artery?** 8. **Is the ultrasound transducer maintaining the artery shape without squeezing it?** 9. **Is the catheter tip (white-dot) visualized on the ultrasound screen?** 10. **Can the participant measure the subcutaneous depth of the radial artery?** | 0 = No 1 = YES  0 = No 1 = YES  0 = No 1 = YES  0 = No 1 = YES  0 = No 1 = YES  0 = No 1 = YES  0 = No 1 = YES  0 = No 1 = YES  0 = No 1 = YES  0 = No 1 = YES |
| **2. Inserting the angiocatheter** | |
| 1. **Did the participant insert the catheter at an angle of approximately 45-60° to the wrist?** 2. **After a flash of blood is noted, did the participant advance and bring down the needle angle to 10-15°?** 3. **Did the participant dynamically position the needle-tip (white dot)?** 4. **Did the participant see the white dot before cannulating the radial artery anterior wall?** 5. **Did the participant puncture the midline of the radial artery anterior wall?** 6. **Was the participant successful to cannulate the radial artery at one’s first attempt?** 7. **Did the participant apply pressure proximal to the puncture site, to prevent loss of blood from hub when the guidewire was removed?** | 0 = No 1 = YES  0 = No 1 = YES  0 = No 1 = YES  0 = No 1 = YES  0 = No 1 = YES  0 = No 1 = YES  0 = No 1 = YES |
| **Total Sum** | **/ 17** |
