# Supplementary material for: Simulation-based training using a vessel phantom effectively improved first attempt success and dynamic needle-tip positioning ability for ultrasound-guided radial artery cannulation in real patients: An assessor-blinded randomized controlled study
Source: PLoS One. 2020 Jun 11;15(6):e0234567. doi: 10.1371/journal.pone.0234567 (PMC7289374; doi:10.1371/journal.pone.0234567)
Supplement: S4 Table — (DOCX) [file pone.0234567.s004.docx]

**Supplement table 4.** The questions that failed to score points on the checklist among successful first attempt cases.

|  |  | Simulation group  (N=36) | |  | Control group  (N=20) | |
| --- | --- | --- | --- | --- | --- | --- |
|  |  | **Yes** | **No** |  | **Yes** | **No** |
| Is the ultrasound transducer maintaining the artery shape without squeezing it? |  | 36 (100) | 0 |  | 19 (95.0) | 1 (5.0) |
| Is the angiocatheter needle-tip (hyperechoic dot) visualized at least once  on the ultrasound screen? |  | 36 (100) | 0 |  | 14 (70.0) | 6 (30.0) |
| Did the participant dynamically position the needle-tip (hyperechoic dot)? |  | 25 (69.4) | 11 (30.6) |  | 3 (15.0) | 17 (85.0) |
| Did the participant see the hyperechoic dot before cannulating  the radial artery anterior wall? |  | 25 (69.4) | 11 (30.6) |  | 8 (40.0) | 12 (60.0) |
| Did the participant puncture the midline of the radial artery anterior wall? |  | 22 (61.1) | 14 (38.9) |  | 4 (20.0) | 16 (80.0) |

Values are presented as numbers (percentile).
