# Supplementary material for: Simulation-based training using a vessel phantom effectively improved first attempt success and dynamic needle-tip positioning ability for ultrasound-guided radial artery cannulation in real patients: An assessor-blinded randomized controlled study
Source: PLoS One. 2020 Jun 11;15(6):e0234567. doi: 10.1371/journal.pone.0234567 (PMC7289374; doi:10.1371/journal.pone.0234567)
Supplement: Attachment — Submitted filename: Response to Reviewers_2.docx [file pone.0234567.s007.docx]

May, 26, 2020

**Simulation-based training using a vessel phantom effectively improved first attempt success and dynamic needle-tip positioning ability for ultrasound-guided radial artery cannulation in real patients: an assessor-blinded randomized controlled study**

**Dear Editor**

We thank you and the reviewers of *Plos One* for taking the time to review our article once again. We made corrections and clarifications to the manuscript to address the reviewer comments. All authors have read and approved the revised manuscript. The changes are summarized below.

**Reviewer #2**

**1. Line 134-135. The authors responded by clarifying the experiences of E.J. Oh but because this is a study evaluating dynamic needle tip positioning, they should also add what the dynamic needle tip positioning experience of this provider was.**

**Response:** As the reviewer suggested we have added information on dynamic needle tip positioning experience of the simulation tutor (E. J. Oh) in our study. The simulation tutor (E. J. Oh) has more than 200 experiences in ultrasound-guided radial artery cannulation using the dynamic needle tip-positioning and have conducted a study on ultrasound-guided vascular cannulation using the dynamic needle-tip positioning technique in children under two year old. Also, each year, the tutor has been teaching dynamic needle tip-positioning skills with verbal assistance and skill demonstration as part of the standard curriculum in our tertiary medical center. We have added more details about this to the revised Methods section as follows:

“The simulation tutor (E. J. Oh) is a skilled anesthesiologist with more than 200 experiences in ultrasound-guided radial artery cannulation using the dynamic needle tip-positioning. In particular, more than half of experiences of E. J. Oh in ultrasound-guided radial artery cannulation using the dynamic needle tip-positioning were done in children under two years old, who are expected to have procedural difficulties. Also, each year, the tutor has been teaching dynamic needle tip-positioning skills with verbal assistance and skill demonstration as part of the standard curriculum in our tertiary medical center.” In lines 133-139.

**2. Line 153-154. Because this is an evaluation of simulation training vs other training methods, it should be clear what the control group experience was. How many procedures on average did they participants in the control group witness? Were these witnessed experiences all dynamic needle tip positioning or not?**

**Response:** We fully agree with your comments. The participants in the control group watched a video clip including operating an ultrasound machine, ultrasound transducer manipulation, and dynamic needle-tip positioning technique. The video clip was provided to all participants in the control group, allowing participants to watch whenever they wanted. In addition, the participants in the control group observed the actual performances of a skilled researcher (E. J. Oh) in more than 10 real patients over a month. We have added the information on proctor experience level to the revised Methods section.

“The participants in the control group watched a video clip including operating an ultrasound machine, ultrasound transducer manipulation, and dynamic needle-tip positioning technique. The video clip was provided to all participants in the control group, allowing participants to watch whenever they wanted over a month. In addition, during this period the participants in the control group also observed the actual ultrasound-guided radial artery cannulation using the dynamic needle tip-positioning by a skilled researcher (E. J. Oh) in more than 10 real patients.” In lines 156-161.

**3. Line 165-166. It seems like this line was edited but both the original and edited sentences remained in the manuscript. Please correct this**

**Response:** We appreciate the reviewer for the attention. As the reviewer pointed out we have erased the duplicated sentence below.

“The cannulation performance of each participant was video recorded.” to “ ” In line 171.

**4. Line 212. "was all comparable" should read "were all comparable"**

**Response:** We have revised the sentence as the reviewer pointed out in the revised Result section.

“Training year, baseline clinical experience of palpated radial artery cannulation, and ultrasound-guided central line cannulation were all comparable between the two groups (Table 1).” In lines 216-218.

**5. Supplement table 4. It is not clear how to read this table. Because these are all questions, maybe it would be better to create "yes" and "no" columns for the control and the simulation group and then fill in the number of responses**

**Response:** As the reviewer suggested we have revised the supplement table 4. Please refer to the attached file < Supplement table 4>.

**6. Line 284. The word "enhance" does not fit in the sentence well. Consider using enhancement, improvement, etc.**

**Response:** We appreciate the reviewer for the comment. We have changed the description in the revised Discussion section.

“Ten simulation sessions were sufficient to demonstrate improvement in skill proficiency, which is in agreement with the findings of previous study suggesting range of six to ten simulation training sessions to demonstrate competence in ultrasound-guided radial artery cannulation.” In lines 288-291.

**7. Line 302. The 50% first pass success rate in novices is not higher than expected. Ueda et al in A randomized controlled trial of radial artery cannulation guided by doppler vs palpation vs ultrasound, achieved a first pass of 53% in anesthesia trainees with < 5 USG radial artery cannulations. levin et al in Use of ultrasound guidance in the insertion of radial artery catheters, Crit Care Med 2003, achieved a first pass success rate of 62% in operators with ultrasound guided central venous catheter experience but novices in radial artery cannulation with ultrasound guidance**

**Response:** We appreciate the reviewer for the comment. We have changed the description in the revised Discussion section according to your comments.

“On the other hand, the residents in the control group showed a similar first attempt success rates of 53% in anesthesia trainees with less than five experiences in ultrasound-guided radial artery cannulation (1) and 62% in anesthetists with ultrasound-guided central vein insertion experience but novice to ultrasound-guided radial artery cannulation.(2) Among successful first attempt cases, the control group showed less success in terms of dynamic needle-tip positioning ability during the procedure, and this group achieved lower overall procedure level scores than the simulation group.” In lines 307-313.

**Reference**

1.	Ueda K, Bayman E, Johnson C, Odum N, Lee JJ. A randomised controlled trial of radial artery cannulation guided by Doppler vs palpation vs ultrasound. Anaesthesia. 2015;70(9):1039-44.

2.	Levin PD, Sheinin O, Gozal Y. Use of ultrasound guidance in the insertion of radial artery catheters. Critical care medicine. 2003;31(2):481-4.

We hope our additionally revised manuscript will better meet your requirements for publication. We thank the editor and the reviewer of *Plos One* once again for their constructive review of our paper.

Warm regards,

Jeong Jin Min, M.D., Ph.D.

Samsung Medical Center,

Sungkyunkwan University School of Medicine
